# Cholinergic receptor nicotinic alpha 5 subunit polymorphisms are associated with smoking cessation success in women

**DOI:** 10.1186/s12881-018-0571-3

**Published:** 2018-04-05

**Authors:** Paulo Roberto Xavier Tomaz, Juliana Rocha Santos, Jaqueline Scholz, Tânia Ogawa Abe, Patrícia Viviane Gaya, André Brooking Negrão, José Eduardo Krieger, Alexandre Costa Pereira, Paulo Caleb Júnior Lima Santos

**Affiliations:** 1 Laboratory of Genetics and Molecular Cardiology, Instituto do Coracao (InCor), Hospital das Clinicas HCFMUSP, Faculdade de Medicina, Universidade de Sao Paulo, Sao Paulo, SP Brazil; 2 Smoking Cessation Program Department, Instituto do Coracao (InCor), Hospital das Clinicas HCFMUSP, Faculdade de Medicina, Universidade de Sao Paulo, Sao Paulo, SP Brazil; 3 Department of Pharmacology, Universidade Federal de Sao Paulo – UNIFESP, Sao Paulo, SP Brazil

**Keywords:** *CHRNA5*, Nicotine dependence, Smoking cessation, Polymorphism

## Abstract

**Background:**

The identification of variants in the nicotinic acetylcholine receptor (nAChR) subunit genes associated with smoking phenotypes are increasingly important for prevention and treatment of nicotine dependence. In the context of personalized medicine, the aims of this study were to evaluate whether cholinergic receptor nicotinic alpha 2 (*CHRNA2)*, cholinergic receptor nicotinic alpha 3 *(CHRNA3)*, cholinergic receptor nicotinic alpha 5 (*CHRNA5)* and cholinergic receptor nicotinic beta 3 (*CHRNB3)* polymorphisms were associated with nicotine dependence severity, and to investigate possible pharmacogenetics markers of smoking cessation treatment.

**Methods:**

This study cohort enrolled 1049 smoking patients who received pharmacological treatment (varenicline, varenicline plus bupropion, bupropion plus/or nicotine replacement therapy). Smoking cessation success was considered for patients who completed 6 months of continuous abstinence. Fagerström test for nicotine dependence (FTND) and Issa situational smoking scores (Issa score) were analyzed for nicotine dependence. *CHRNA2* (rs2472553), *CHRNA3* (rs1051730), *CHRNA5* (rs16969968 and rs2036527) and *CHRNB3* (rs6474413) polymorphisms were genotyped by high resolution melting analysis.

**Results:**

Females with GA and AA genotypes for *CHRNA5* rs16969968 and rs2036527 polymorphisms had higher success rate in smoking cessation treatment: 44.0% and 56.3% (rs16969968), 41.5% and 56.5% (rs2036527), respectively, compared with carriers of the GG genotypes: 35.7% (rs16969968), 34.8% (rs2036527), (*P* = 0.03, *n* = 389; *P* = 0.01, *n* = 391). The GA or AA genotypes for the rs16969968 and rs2036527 were associated with higher odds ratio for success in women (OR = 1.63; 95% CI = 1.04 to 2.54; *P* = 0.03 and OR = 1.59, 95% CI = 1.02 to 2.48; *P* = 0.04; respectively). We did not find association of these polymorphisms with nicotine dependence related scores. Polymorphisms in the *CHRNA2*, *CHRNA3* and *CHRNB3* genes were not associated with the phenotypes studied.

**Conclusion:**

*CHRNA5* rs16969968 and rs2036527 were associated with higher success rate in the smoking cessation treatment in women. These findings might contribute to advances in personalized medicine.

**Electronic supplementary material:**

The online version of this article (10.1186/s12881-018-0571-3) contains supplementary material, which is available to authorized users.

## Background

Smoking is a serious public health problem, contributing significantly to the risk of death from cancers, cardiovascular diseases, lung disorders and stroke. Public health agencies aim to reduce tobacco use; however, relapse is common during smoking treatment programs. According to the World Health Organization, if current trends continue, the annual number of deaths from diseases related to smoking could double from five million in 2000 to 10 million in 2020 [1, 2].

Studies have shown that although smoke toxicity is related to many components of the cigarette, nicotine is responsible for the development of dependence [3, 4]. Smokers in Brazil who want to quit have the following first-line pharmacological treatments: nicotine replacement therapy (NRT), bupropion (an inhibitor of the reuptake of norepinephrine, dopamine and nicotinic antagonist) and, varenicline (a partial agonist of nicotinic acetylcholine receptor - nAChR - α4β2) [5–7].

Cigarette smoking is classified as a complex behavior comprising multiple stages such as initiation, experimentation, regular use, addiction, cessation and relapse [8, 9]. To better understand the role played by genes in those stages, twin studies, and allelic association studies of candidate genes and large genome wide-association studies are increasingly required [8–10].

In the last ten years, several studies have identified *loci*, mainly in chromosome 8 and 15, which are highly involved in the pathogenesis of nicotine dependence (ND) process. These regions of the genome include genes encoding various subunits of the nAChR. These receptors are expressed in the brain and are thought to be responsible for mediating the addictive effects of nicotine [1, 8, 10–14]. Therefore, studies have shown the association of polymorphisms in genes encoding the subunits of nicotinic receptors (*CHRNA2*, *CHRNA3*, *CHRNA5*, *CHRNB3*) with the number of cigarettes per day, ND and risk for developing lung cancer [1, 8, 13, 15, 16].

In the context of personalized medicine, the aims of the present study were to evaluate whether *CHRNA2, CHRNA3, CHRNA5* and, *CHRNB3* polymorphisms were associated with level of dependency and, response to smoking cessation therapies in patients from a smoking cessation assistance program.

## Methods

### Study population

The study sample included 1049 smoking patients from PAF (Programa de Assistência ao Fumante /Smoker Assistance Program), Heart Institute (InCor), University of Sao Paulo Medical School, Sao Paulo, Brazil, between January, 2007 and November, 2014. The study protocol was approved by the Institutional Ethics Committee (Comissão de Ética para Análises de Projetos de Pesquisa - CAPPesq − 4024/14/004), and written informed consent was obtained from all participants prior to entering the study. Data from patients who had outcome in smoking treatment were obtained from the PAF database.

The PAF protocol consists of an initial medical visit plus an average of 4 follow-up medical visits for 12 weeks. The follow-up was made by phone in patients who did not continue to come on scheduled medical visits. Clinical data and end-expiratory exhaled carbon monoxide (CO) were collected in all visits. Data about demographic, socio-economic, clinical data and previously diagnosed diseases were acquired at the consult. During clinical anamnesis patients were questioned whether they were undergoing treatment or had a diagnosis of depression and/or anxiety. Patients received behavioral counseling and drug treatment from physicians specialized in smoking cessation. Bupropion plus/or NRT was prescribed for patients who smoked less than 1 cigarette pack per day. Varenicline was prescribed for patients who smoked 1 or more cigarette pack(s) per day, or who failed in previous attempts with bupropion plus/or NRT. The decision to start co-administration of bupropion and varenicline was made if the patient was unable to quit smoking after 2 or 3 months of starting varenicline use, or if the patient stopped smoking, but presented moderate or intense discomfort abstinence symptoms. Continuous abstinence (CA) was investigated after 6 months of starting pharmacotherapy. Smoking status (outcome) was divided into: success group (patients who completed 6 months of CA confirmed by end-expiratory exhaled carbon monoxide < 4 ppm), relapse group (patients who did not complete 6 months of CA), and resistant group (patients who never achieved CA after starting drug treatment) [17, 18].

### FTND and Issa scores

The Fagerström Test for Nicotine Dependence (FTND) and the Issa Situational Smoking Score (Issa score) were used to assess smoking behavior. The FTND is comprised of six questions with different weights for each question, generating a score ranging from 0 to 10 points and individuals are grouped into five categories: 1–2 points = very low dependence; 3–4 points = low dependence; 5 points = medium dependence; 6–7 points = high dependence; and 8–10 points = very high dependence [19]. The Issa score is comprised of four questions, one point for each affirmative answer, generating a scores ranging from 0 to 4 points. Individuals are grouped into categories: 1 point = low dependence; 2–3 points = medium dependence; 4 points = high dependence [20].

The FTND is a revised version of the Fagerström Tolerance Questionnaire (FTQ) and it is based in the cigarette consumption, while the Issa score is based on the psychoactive effects of nicotine on cognitive processes, attention, concentration, mood, well-being and pleasure [20]. The Issa score is used at the PAF to reclassify nicotine dependent patients who achieved a FTND score below 5 but have otherwise considerable ND level in relation to the psychoactive effects of nicotine. As consequences of the psychoactive action of nicotine, smokers can have behaviors that may indicate the intensity of addiction [20].

### Genotyping

The criteria for selection of these polymorphisms was the number of significant replications in genetic association studies.

Genomic deoxyribonucleic acid (DNA) from subjects was extracted from peripheral blood following a standard salting-out procedure. Genotyping for the *CHRNA2* rs2472553 (c.674C > T), *CHRNA3* rs1051730 (c.645C > T), *CHRNA5* rs16969968 (c.1192G > A), *CHRNA5* rs2036527 (g.78559273G > A) and *CHRNB3* rs6474413 (g.42695921G > A) were performed by polymerase chain reaction (PCR) followed by high resolution melting (HRM) analysis according with previous studies [21, 22]. Amplification of the fragment for the *CHRNA2* rs2472553, *CHRNA3* rs1051730, *CHRNA5* rs16969968, *CHRNA5* rs2036527 and *CHRNB3* rs6474413 were performed using the primers shown in Additional file 1: Table S1. As an example, Additional fil 2: Figure S1 shows normalized fluorescence by temperature graphs for the *CHRNA5* rs16969968 polymorphism. Nine percent of the samples were randomly selected and reanalyzed as quality controls and gave identical results.

### Statistical analysis

Continuous variables are presented as mean and standard deviation and categorical variables as frequencies. Chi-square test was performed for the comparative analysis of treatment outcome in the overall group of patients and groups of women and men according to the polymorphisms in *CHRNA2, CHRNA3, CHRNA5* and *CHRNB3* genes. The analysis of the Hardy-Weinberg equilibrium (HWE) was also performed using the chi-square test. The Student’s t-test was used to compare values of the FTND score according to polymorphisms and also, to compare general and clinical characteristics between men and women. Linear regression models were conducted to evaluate the influence of polymorphisms in FTND in the presence of covariates: age, gender (male), race/self-declared color (white) and educational status. Logistic regression multivariate models were used to evaluate the odds ratio (OR) for success (versus relapse plus resistant) according to the polymorphisms. Covariates used in the models were: age, race/self-declared color (white), FTND score and drug group. Studies have tested additive effects, including some of the largest genetic studies on smoking behavior [23] and cessation [24]. However, the dominant model was chosen based on previous studies: *CHRNA2* rs2472553 (CC vs CT + TT) [13], *CHRNA3* rs1051730 (CC vs CT + TT) [25], *CHRNA5* rs16969968 (GG vs GA + AA) [26], *CHRNA5* rs2036527 (GG vs GA + AA) [27] and *CHRNB3* rs6474413 (GG vs GA + AA) [28]. Statistical analyses were carried out using the SPSS software (v.16.0), with the level of significance set at *p* ≤ 0.05.

## Results

### General, clinical, and genetic characteristics of patients

The clinical and demographic characteristics of the 1049 participants are shown in Table 1. Patients in the analysis had a mean age of 54.0 ± 15.0 years and 54.7% were female. Race/color self-declared white was 65.8%. The non-white was composed by patients self-declared as blacks (7.2%) and intermediates (23.9%). In addition, Asians (3.0%) and Amerindians (0.1%) were enrolled. The minor allele frequencies and genotypic distributions for the rs2472553, rs1051730, rs16969968, rs2036527 and rs6474413 were in accordance with Hardy–Weinberg equilibrium (Additional file 1 Table S2).

**Table 1:** Clinical and demographic characteristics of patients undergoing smoking cessation (*n* = 1049)

	Women (*n* = 574)	Men (*n* = 475)	*P* value
Age (years)	54 ± 18	54 ± 11	0.52
Race/color self-declared, White (%)	62.2	69.3	0.08
Body mass index (Kg/m^2^)	27 ± 6	27 ± 5	0.17
Educational status, college (%)	28.4	32.4	0.44
Hypertension (%)	45.1	53.7	0.006
Coronary artery disease (%)	14.1	26.9	< 0.001
Acute myocardial infarction (%)	14.5	31.2	< 0.001
Dyslipidemia (%)	40.6	46.1	0.07
Diabetes mellitus type 2 (%)	14.3	17.5	0.16
Depression (%)	24.7	13.7	< 0.001
Anxiety (%)	21.6	15.8	0.02
Obstructive pulmonar chronic disease (%)	16.9	15.4	0.50
Asthma (%)	1.6	1.1	0.47

### Success rate in smoking cessation according to genotypes

Table 2 shows the success rate in smoking cessation for patients according to the genotypes for *CHRNA2* rs2472553, *CHRNA3* rs1051730, *CHRNA5* rs16969968 and rs2036527 and *CHRNB3* rs6474413 polymorphisms. The GA and AA genotypes for the *CHRNA5* rs2036527 and rs16969968 polymorphisms were associated with higher rates of success in smoking cessation treatment in the female group (*n* = 389, *n* = 391); (*P* = 0.03, *P* = 0.01), respectively. The frequencies of success according to genotypes among the drug treatment groups were not different. We found no association of *CHRNA2* rs2472553, *CHRNA3* rs1051730 and *CHRNB3* rs6474413 polymorphisms with treatment outcome. The *CHRNA2* rs2472553, *CHRNA3* rs1051730 and *CHRNB3* rs6474413 polymorphisms showed the following OR for success: 1.17 (95% CI = 0.81–1.69, *p* = 0.42); 1.02 (95% CI = 0.75–1.40, *p* = 0.88); and 1.00 (95% CI = 0.57–1.75, *p* = 1.00), respectively.

**Table 2:** Success rate in smoking cessation for patients according to genotypes

	*CHRNA2* rs2472553			
Patient groups	Success rate (%)			
	CC	CT	TT	*P* value
Overall group (*n* = 737)	44.7	47.5	46.2	0.81
Women (*n* = 395)	40.6	41.2	71.4	0.26
Men (*n* = 342)	49.4	54.5	16.7	0.19
*CHRNA3* rs1051730				
Patient groups	Success rate (%)			
	CC	CT	TT	P value
Overall group (*n* = 742)	44.8	45.6	50.6	0.64
Women (*n* = 396)	36.6	43.5	51.0	0.14
Men (*n* = 346)	54.3	47.9	50.0	0.52
*CHRNA5* rs16969968				
Patient groups	Success rate (%)			
	GG	GA	AA	P value
Overall group (*n* = 731)	43.0	46.6	54.3	0.18
Women (*n* = 389)	35.7	44.0	56.3	0.03
Men (*n* = 342)	52.0	49.1	51.5	0.87
*CHRNA5* rs2036527				
Patient groups	Success rate (%)			
	GG	GA	AA	P value
Overall group (*n* = 734)	43.9	44.4	55.0	0.13
Women (*n* = 391)	34.8	41.5	56.5	0.01
Men (*n* = 343)	53.8	47.5	52.6	0.53
*CHRNB3* rs6474413				
Patient groups	Success rate (%)			
	GG	GA	AA	P value
Overall group (*n* = 727)	46.0	46.0	45.9	1.00
Women (*n* = 388)	41.7	44.4	39.5	0.64
Men (*n* = 339)	51.9	47.8	52.8	0.67

Table 3 shows a multivariate logistic regression analysis for the success in smoking cessation according to the polymorphisms in *CHRNA5* gene in women. The GA or AA genotypes for *CHRNA5* rs16969968 and rs2036527 were associated with higher OR for success in women (OR = 1.63; 95% CI = 1.04 to 2.54; *P* = 0.03) (OR = 1.59; 95% CI = 1.02 to 2.48; *P* = 0.04), respectively. Multivariate models with the inclusion of variables were: age, race/color, FTND score and drug group.

**Table 3:** Multivariate logistic regression analysis for success in smoking cessation according to the *CHRNA5* rs16969968 (*n* = 389) and rs2036527 (*n* = 391) polymorphisms in women

	OR	95% CI	*P* value
Genotypes GA or AA for *CHRNA5* rs16969968	1.63	1.04–2.54	0.03
Age	0.99	0.98–1.01	0.34
Race/color self-declared, White	1.20	0.76–1.91	0.43
FTND score	0.96	0.88–1.05	0.35
Drug group	1.01	0.83–1.25	0.86
	OR	95% CI	P value
Genotypes GA or AA for *CHRNA5* rs2036527	1.59	1.02–2.48	0.04
Age	0.99	0.97–1.01	0.33
Race/color self-declared, White	1.28	0.82–2.00	0.29
FTND score	0.97	0.87–1.05	0.44
Drug group	1.01	0.83–1.24	0.93

*FTND* Fagerström test for nicotine dependence

A stratified analysis by self-reported race showed the following odds ratios for white women (OR = 1.67; 95% CI = 1.06 to 2.43; *P* = 0.03 and OR = 1.64; 95% CI = 1.01 to 2.73; *P* = 0.04) and for non-white women (OR = 1.54; 95% CI = 1.03 to 3.23; *P* = 0.04 and OR = 2.06; 95% CI = 0.99 to 4.28; *P* = 0.05), respectively. Males and females were analyzed together with the genotypes by sex interaction, but the finding was not significant.

Women showed a higher incidence of both depression and anxiety than men (69.0% vs. 31.0%, *P* = 0.001; 63.8% vs. 36.2%, *P* = 0.03, respectively). Given these differences, the variables depression and/or anxiety were tested in logistic regression. These variables were not associated with treatment success in this model. However, GA and AA genotypes for rs2036527 and rs16969968 polymorphisms remained significantly associated.

### FTND score according to CHRNA2, CHRNA3, CHRNA5, CHRNB3 polymorphisms

We did not observe significant differences in the FTND score according to *CHRNA2* rs2472553, *CHRNA3* rs1051730, *CHRNA5* rs16969968, *CHRNA5* rs2036527 and *CHRNB3* rs6474413 polymorphisms in the overall patient group (*n* = 899) (Additional file 1: Table S3). In addition, studied polymorphisms were not associated with FTND score in multiple linear regression models (Additional file 1: Table S4).

## Discussion

The main findings of this study was the association of GA and AA genotypes for both *CHRNA5* rs16969968 and rs2036527 polymorphisms with increased success rate in smoking cessation treatment in women. Many studies have identified the association of polymorphisms in the *CHRNA5-A3-B4* gene with smoking cessation in response to smoking cessation therapies [2, 29–31]. Recently, our group of researchers found the association of the *CHRNA4* rs10443196 and cytochrome P450 2B6 (*CYP2B6)* 785A > G polymorphisms with a higher success rate in varenicline and bupropion treatment, respectively [32, 33].

The association of the *CHRNA5* rs16969968 polymorphism with higher rates of success in smoking cessation treatment in women is corroborated by previous studies. As shown in vitro experiments Bierut et al. [14] and Kuryatov et al. [34], individuals carrying the allele A may express nAChR subtypes composed by α5 subunits with reduced function. Bergen et al. [35] showed that a lower frequency of the allele for rs1051730, which is in strong linkage disequilibrium with rs16969968, was associated with a higher withdrawal rate with NRT. Bergen et al. pointed out that smokers with reduced function of the α5 subunit and associated with increased ND may have greater difficulty quitting. However, they suggested that the prescription of NRT can improve cognitive performance in smoking abstinence, maintaining the normal functioning of the brain after quitting, and this effect may be stronger for individuals with the rs1051730 risk allele. Similarly, Chen et al. [36] reported that individuals with the AA genotype for the rs16969968 had higher withdrawal rate in the treatment with NRT. In the study by Jensen et al. [37], the A allele was associated with lower rates of aversive effects to the nicotine and also with improvement in cognitive control. Jensen et al. suggested that the A allele may be important in tolerability and in response to treatments with NRT. They added that, remarkably, the α5 subunit plays an essential role in mediating aversive effects to the nicotine. Interestingly, King et al. [31] showed the association of others risk alleles for ND in *locus* chr15q25 with lower incidence of nausea. The authors suggest that this may be explained by the tolerance, because individuals with higher ND have higher daily intake of nicotine, are more tolerant and therefore are less likely to experience nausea in response to a nicotinic partial agonist such as varenicline. Therefore, reduced function of the α5 subunit, the higher tolerability of NRT and varenicline and improvement of cognitive control may be possible explanations for the association of rs16969968 with success in this study. One other possibility is that the rs16969968 can be in linkage disequilibrium with other functional polymorphisms that could be involved in smoking cessation process and/or mechanisms in response to the NRT.

There was a higher frequency of depression and/or anxiety in the women’s group, which according to some studies could reduce success rates in the smoking cessation treatment [38–42]. Zawertailo et al. [38] conducted a study of 13,000 smokers and found that individuals with recurrent depression were significantly less likely to quit smoking compared to those with no history of depression. Goodwin et al. [39] found that panic attacks and social anxiety disorder were more likely in female smokers than male smokers. Stepankova et al. [40], in a study of 1730 smokers, found that 289 had a history of depression. The tobacco abstinence rate at 1 year was 32.5% for smokers with a history of depression and 38.7% for those with no history. In our study, although the frequencies of depression and anxiety were higher in women than in men, these variables were not associated with treatment outcome and rs16969968 and rs2036527 polymorphisms remained significantly associated with smoking cessation in a multivariate model.

There are evidences that gender can be an important factor in smoking cessation process. Beltz et al. [43], evaluating functional magnetic resonance imaging and behavioral data from 50 adult daily smokers (23 women), found that women had an increased nicotine tolerance when compared to men. Smith et al. [44] found that women were 31% less likely to quit smoking than men. Pierce et al. [45] reported significant sex differences in smoking cessation. They observed the women had weight gain, higher prevalence of depression disorders and increased need of social support to stop smoking.

Some studies showed that several factors may influence sex/gender differences [46], such as biopsychosocial factors, menstrual cycle, hormonal [47, 48], having children or not, and smoking cessation medication [44, 49].

In the present study, we found no association of *CHRNA2* rs2472553, *CHRNA3* rs1051730 and *CHRNB3* rs6474413 polymorphisms with treatment outcome. The results of our study corroborate the findings of previous studies. Ruyck et al. [50] and Sarginson et al. [2] found no association for rs1051730 with outcome of treatment. However, Munafo et al. [51] identified the T allele associated with decreased likelihood of smoking cessation. Bergen et al. [35] associated the T allele with higher withdrawal rates with NRT and lower withdrawal rate in placebo treatment.

Regarding FTND score, several studies have identified the association of polymorphisms in genes encoding nAChR alpha and beta subunits with FTND and, number of cigarettes per day [52–57]. However, our study found no significant association of the polymorphisms studied with FTND, consistent with the findings of previous studies, which found no association of *CHRNA3* rs1051730, *CHRNA5* rs16969968, *CHRNA5* rs2036527 or *CHRNA5* rs6474413 polymorphisms with phenotypes related to smoking, including ND [58–62].

Etter et al. [62] suggested that the reason for missing association of polymorphisms studied, including rs16969968, can be explained by the difference in the methods used, or due to the small sample size. Amos et al. [61] did not find association of rs16969968 polymorphism with smoking behavior in either African American case patients or African American control participants. The authors point out some limitations of no association with the studied phenotypes, as the sample size limited and also because individuals come from a single center; it is possible that some degree of population structure influenced the findings.

There are some limitations in our study that should be mentioned. First, most patients treated at the PAF were classified as moderately or highly dependent, which may have affected the analysis of association between genotypes and FTND score. Second, the race/color of patients was self-report and we did not performed genetic ancestry. Third, we did not perform the correction of *P* values for multiple testing. Fourth, besides the available variables used in the multivariate models other relevant factors that could be important, such as the functionality of the receptors, motivation to quit smoking, biopsychosocial and others factors related to sex/gender, could have influenced the results.

## Conclusion

We showed that *CHRNA5* rs16969968 and rs2036527 polymorphisms were associated with higher rates of success in smoking cessation treatment in the female group. These results might contribute to advances in personalized medicine.

## Additional files

Additional file 1: **Table S1.** included: polymorphisms, primer F, primer R and base pairs. (DOCX 22 kb)

Additional file 2: **Figure S1.** Graphs of the *CHRNA5* rs16969968 genotyping. Nucleotide changes results in different curve patterns using high resolution melting analysis. A: Graph of normalized fluorescence by temperature. B: Graph of normalized fluorescence (based on genotype 2) by temperature. 1 = GG, 2 = GA, 3 = AA. (DOCX 114 kb)

## Abbreviations

CA: Continuous abstinence
CAPPesq: Comissão de Ética para Análises de Projetos de Pesquisa
*CHRNA2*: cholinergic receptor nicotinic alpha 2
*CHRNA3*: cholinergic receptor nicotinic alpha 3
*CHRNA5*: cholinergic receptor nicotinic alpha 5
*CHRNB3*: cholinergic receptor nicotinic beta 3
CO: End-expiratory exhaled carbon monoxide
*CYP2B6*: cytochrome P450 2B6
DNA: deoxyribonucleic acid
FTND: Fagerström test for nicotine dependence
FTQ: Fagerström tolerance questionnaire
HRM: high resolution melting
HWE: Hardy-Weinberg equilibrium
InCor: Heart Institute
Issa score: Issa situational smoking scores
nAChR: Nicotinic acetylcholine receptor
ND: nicotine dependence
NRT: Nicotine replacement therapy
OR: odds ratio
PAF: Programa de Assistência ao Fumante /Smoker Assistance Program
PCR: polymerase chain reaction
