# Supplementary material for: Cholinergic receptor nicotinic alpha 5 subunit polymorphisms are associated with smoking cessation success in women
Source: BMC Med Genet. 2018 Apr 5;19:55. doi: 10.1186/s12881-018-0571-3 (PMC5887212; doi:10.1186/s12881-018-0571-3)
Supplement: Supplementary file 1 — Table S1. included: polymorphisms, primer F, primer R and base pairs. (DOCX 22 kb) [file 12881_2018_571_MOESM1_ESM.docx]

**SUPPLEMENTARY MATERIAL**

**Supplementary table 1.** Primers description

| **Polymorphisms** | **Primer F** | **Primer R** | ***Base pairs*** |
| --- | --- | --- | --- |
| *CHRNA2* rs2472553 | CTGTGTTCCTGTCCTTCACAAA | TGACAGGTGACAAACACTCACC | 78 |
| *CHRNA3* rs1051730 | ACCTCAAGGACTATTGGGAGAG | GCAGTTGTACTTGATGTCGTGTT | 80 |
| *CHRNA5* rs16969968 | CTTCTAGAAACACATTGGAAGCTG | TGATGTGTCTTGTAATGTAGCGAAT | 60 |
| *CHRNA5* rs2036527 | AGACAGCTGTGGAACTGGAAAT | ATTCTAAGCCACCTGATCCAAA | 71 |
| *CHRNB3* rs6474413 | TGAAAATAAAGGTGAAACTTCCTG | AATGGTCTCAGGAGTAAATCAGC | 66 |

**Supplementary table 2.** Genotypic frequencies and Hardy-Weinberg equilibrium analysis

| ***CHRNA2* rs2472553** | ***CHRNA3***  **rs1051730** | ***CHRNA5* rs16969968** | ***CHRNA5* rs2036527** | ***CHRNB3* rs6474413** |
| --- | --- | --- | --- | --- |
| **Genotypic distribution (%)** | | | | |
| CC= 76.2 (n=776) | CC= 42.1 (n=431) | GG= 43.0 (n=435) | GG= 39.6 (n=402) | GG= 8.3 (n=83) |
| CT= 21.9 (n=223) | CT= 45.6 (n=467) | GA= 44.9 (n=454) | GA= 46.0 (n=466) | GA= 42.4 (n=427) |
| TT= 1.9 (n=19) | TT= 12.3 (n=126) | AA= 12.1 (n=123) | AA= 14.4 (n=146) | AA= 49.3 (n=496) |
| **Minor allele (%)** | | | | |
| T Allele = 12.8 | T Allele = 35.1 | A Allele = 34.6 | A Allele = 37.4 | G Allele = 29.5 |
| **HWE** | | | | |
| X^2^ = 0.41 | X^2^ = 0.0008 | X^2^ = 0.07 | X^2^ = 0.34 | X^2^ = 0.44 |
| P value = 0.52 | P value = 0.98 | P value = 0.79 | P value = 0.56 | P value = 0.51 |

Minor allele found in the present study (patients from the Smoker Assistance Program - PAF). n = sample size. HWE = Hardy-Weinberg equilibrium. If P <0.05 not consistent with HWE.

**Supplementary table 3.** Results of patients to FTND score

|  | **Genotypes** | **Mean** | **95% CI** | **p value** |
| --- | --- | --- | --- | --- |
| ***CHRNA2* rs2472553** | CC (n=686) | 6.43 | (6.25 – 6.61) | 0.36 |
|  | CT + TT (n=213) | 6.57 | (6.25 – 6.90) |  |
| ***CHRNA3* rs1051730** | CC (n=378) | 6.32 | (6.07 – 6.58) | 0.40 |
|  | CT + TT (n=521) | 6.56 | (6.36 – 6.77) |  |
| ***CHRNA5* rs16969968** | GG (n=390) | 6.36 | (6.11 – 6.60) | 0.45 |
|  | GA + AA (n=509) | 6.54 | (6.33 – 6.75) |  |
| ***CHRNA5* rs2036527** | GG (n=357) | 6.27 | (6.01 – 6.53) | 0.21 |
|  | GA + AA (n=542) | 6.59 | (6.39 – 6.79) |  |
| ***CHRNB3* rs6474413** | GG (n=74) | 6.54 | (5.95 – 7.13) | 0.82 |
|  | GA + AA (n=825) | 6.46 | (6.29 – 6.62) |  |

95% CI = 95% confidence interval.

**Supplementary table 4.** Variables of the multiple linear regression model for FTND score

| **Variable** | **β coefficient**  **(standard error)** | **p value** |
| --- | --- | --- |
| **FTND** | | |
| **Number of variant allele for the *CHRNA2* rs2472553** | 0.09 (0.2) | 0.62 |
| **Age** | 0.002 (0.005) | 0.71 |
| **Sex (male)** | -0.01 (0.2) | 0.95 |
| **Race/color self-declared (White)** | 0.1 (0.2) | 0.48 |
| **Scholarity** | 0.09 (0.1) | 0.38 |
| **FTND** | | |
| **Number of variant allele for the *CHRNA3* rs1051730** | 0.08 (0.1) | 0.54 |
| **Age** | 0.002 (0.005) | 0.69 |
| **Sex (male)** | -0.009 (0.2) | 0.96 |
| **Race/color self-declared (White)** | 0.1 (0.2) | 0.58 |
| **Scholarity** | 0.09 (0.1) | 0.40 |
| **FTND** | | |
| **Number of variant allele for the *CHRNA5* rs16969968** | 0.08 (0.1) | 0.52 |
| **Age** | 0.002 (0.005) | 0.71 |
| **Sex (male)** | 0.01 (0.2) | 0.94 |
| **Race/color self-declared (White)** | 0.1 (0.2) | 0.46 |
| **Scholarity** | 0.08 (0.1) | 0.49 |
| **FTND** | | |
| **Number of variant allele for the *CHRNA5* rs2036527** | 0.2 (0.1) | 0.18 |
| **Age** | 0.002 (0.005) | 0.72 |
| **Sex (male)** | -0.02 (0.2) | 0.91 |
| **Race/color self-declared (White)** | 0.07 (0.2) | 0.74 |
| **Scholarity** | 0.08 (0.1) | 0.48 |
| **FTND** | | |
| **Number of variant allele for the *CHRNB3* rs6474413** | 0.02 (0.1) | 0.90 |
| **Age** | 0.003 (0.005) | 0.60 |
| **Sex (male)** | -0.05 (0.2) | 0.77 |
| **Race/color self-declared (White)** | 0.1 (0.2) | 0.59 |
| **Scholarity** | 0.1 (0.1) | 0.31 |

Numbers of variant allele for *CHRNA2* rs2472553 were 0, 1 or 2 for CC, CT, or TT, respectively.

For C*HRNA3* rs1051730 were 0, 1 or 2 for CC, CT or TT, respectively.

For *CHRNA5* rs16969968 were 0, 1 or 2 for GG, GA or AA, respectively.

For *CHRNA5* rs2036527 were 0, 1 or 2 for GG, GA or AA, respectively.

For *CHRNB3* rs6474413 were 0, 1 or 2 for GG, GA, or AA, respectively.

FTND = *Fagerström test for nicotine dependence* (n=976).
