# Supplementary figures and images for: Cholinergic receptor nicotinic alpha 5 subunit polymorphisms are associated with smoking cessation success in women
Source: BMC Med Genet. 2018 Apr 5;19:55. doi: 10.1186/s12881-018-0571-3 (PMC5887212; doi:10.1186/s12881-018-0571-3)

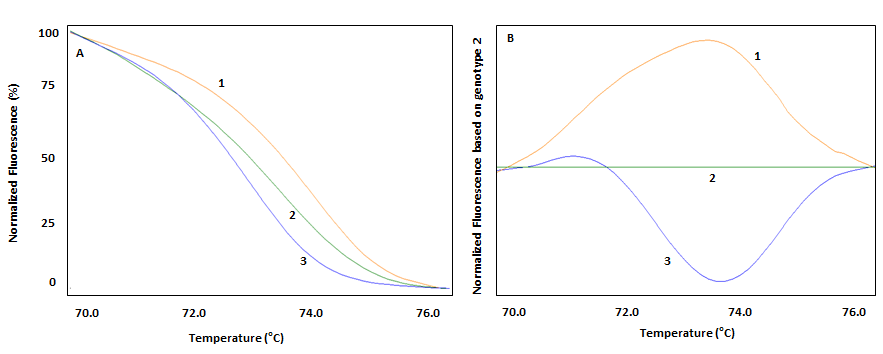


Additional file 2

Supplement: Supplementary file 2 — Figure S1. Graphs of the CHRNA5 rs16969968 genotyping. Nucleotide changes results in different curve patterns using high resolution melting analysis. A: Graph of normalized fluorescence by temperature. B: Graph of normalized fluorescence (based on genotype 2) by temperature. 1 = GG, 2 = GA, 3 = AA. (DOCX 114 kb) [file 12881_2018_571_MOESM2_ESM.docx]
